# How often should self-monitoring of blood pressure be repeated? A secondary analysis of data from two randomized controlled trials

**DOI:** 10.1097/HJH.0000000000004123

**Published:** 2025-08-20

**Authors:** Frances Rose, Richard S. Stevens, Kate S. Morton, Lucy Yardley, Richard J. McManus

**Affiliations:** a Nuffield Department of Primary Care Health Sciences, University of Oxford, Oxford; b Department of Health Sciences, University of York, York; c School of Psychological Science, University of Bristol, Bristol and School of Psychology, University of Southampton, Southampton; d Brighton and Sussex Medical School, University of Brighton and University of Sussex, Brighton, UK

**Keywords:** blood pressure, frequency, home monitoring, self-monitoring

## Abstract

**Background:**

Little evidence exists around the optimal frequency of self-monitoring of blood pressure (BP). Testing too frequently can lead to erroneous management changes due to random “noise” leading to raised measurements by chance. This study used recent trial data to evaluate self-monitored BP over time, aiming to determine how frequently patients should self-monitor.

**Methods:**

Data from patients with home BP ≤135/85 mmHg on stable medication in the self-monitoring groups of two trials were analysed using a mixed effects model. The primary outcome was mean change in BP per month. Secondary outcomes included intra-individual BP variability, and probability of truly raised BP over time.

**Results:**

232 participants from HOMEBP, and 582 participants from TASMINH4 were included. The mean changes in systolic BP per month per study were −0.1 mmHg [standard deviation (SD) 0.6 mmHg], and −0.2mmHg [SD 0.7 mmHg], respectively. Intra-individual systolic variability (SD) per month was 4.7 and 5.1 mmHg respectively. Using TASMINH4 data, from a starting systolic BP of 130 mmHg, re-testing BP after 6 months resulted in a probability of 18% that BP ≥135 mmHg, with a 25% probability that this reflected truly raised BP; after 12 months the probability of a raised reading was 26% with a 65% probability this reflected a true rise.

**Conclusions:**

In the absence of medication changes, there was very little change in mean self-monitored BP per month, with larger variability within an individual's monthly submitted readings. For people with initially controlled BP and stable medication, repeating self-monitoring at 12 months is likely to be appropriate in guiding management.

## INTRODUCTION

High blood pressure (BP) is a leading risk factor for cardiovascular disease. In the UK, more than 50% of people aged 65 or over have a BP greater than 140/90 mmHg, despite the availability of effective treatments and widespread understanding that lowering BP reduces morbidity and mortality [1]. Self-monitoring, where individuals measure their own BP, usually in a home environment, can improve BP control and is increasingly being used as part of routine hypertension management in primary care settings [2,3]. Self-monitoring is used to guide titration when BP rises above a certain threshold. However, there is considerable variability in how such monitoring is implemented, and there is currently little evidence on which to base guidance around the optimal frequency to undertake measurements to inform management.

An individual's BP undergoes continuous dynamic fluctuations, due to environmental, physical and emotional factors, as well as regulatory mechanisms of the cardiovascular system to maintain adequate organ perfusion [4]. Variation can also be affected by error and imprecision, including due to the operator (clinician or patient) error, and measurement imprecision (the systematic and random error not attributed to true changes in the measured parameter) [5]. Whilst variability in BP has been associated with a small increase in cardiovascular and mortality outcomes, separate from the effect of mean blood pressure [6], it can also make it more challenging to detect “true” changes in baseline BP which confer greater cardiovascular risk to the individual. This is often referred to as trying to differentiate a “signal” (i.e. a true increase in BP) from the background “noise” created by this expected variability (i.e. false positives) [7]. In order to appropriately treat patients, clinicians need to determine whether changes seen on home monitoring are demonstrating signal or noise [8]. Inappropriately frequent repeat testing of patient's BP can lead to misinterpretation of apparent increases, which are, in reality, false positive results reflecting measurement variability [9]. Therefore, higher frequency of testing does not necessarily lead to improved clinical management and risk reduction, and comes with higher financial costs to the healthcare system and higher personal costs to individual patients [10,11]. This has been shown in other chronic conditions, such as HbA1c testing for diabetes, where the true biological rate of change is actually much smaller than the total variability in results of monitoring [12]. This highlights the need for better understanding of, and an increase in the evidence around, chronic disease monitoring in primary care.

A previous evaluation used clinic readings from the treatment arm of the perindopril protection against recurrent stroke trial (PROGRESS) to examine how variability in blood pressure changes over time [9]. This showed, for people with controlled blood pressure at baseline, only a 50% probability that an increase in clinic blood pressure readings reflected a true blood pressure rise at two years after enrolment, but similar analyses have not been replicated with self-monitored BP. There have now been several large trials into self-monitoring of blood pressure, where participants on treatment for hypertension submit frequent home BP readings for at least a year [2]. These trials provide an opportunity to examine changes in self-monitored BP over time, including variability within reported monthly readings and month by month trends, both for individuals and across study populations. The aim of this study was to determine how frequently patients with controlled treated hypertension should self-monitor to maintain blood pressure to reduce the risk of misclassification of BP as being above threshold.

## METHODS

### Data sources

Data from the self-monitoring arms of the HOMEBP and TASMINH4 trials were examined [13,14]. HOMEBP [13] was a randomized controlled trial evaluating a digital intervention which involved self-monitoring of blood pressure. It included 622 participants with hypertension, recruited from 76 general practices, allocated to either self-monitoring with a digital intervention (305 participants) or usual care groups (317 participants). Each month participants in the self-monitoring arm submitted seven consecutive days of home BP readings. If mean home BP was controlled for three consecutive months (defined as 100–134/≤84 mmHg diastolic), patients reduced self-monitoring frequency to once every 8 weeks, but with monitoring reverting back to monthly if mean blood pressure subsequently rose ≥135/85 mmHg. Data were available from 271 participants in the self-monitoring arm over one year, which were considered for this analysis.

In TASMINH4 [14] patients with hypertension (BP >140/90mmHg) were randomized to usual care (394 participants), self-monitoring alone (391 participants) or self-monitoring with telemonitoring (389 participants). For those undertaking self-monitoring only, they were provided with a colour chart to help understand their BP readings, and guide them regarding appropriate management. Participants in the telemonitoring group sent readings via SMS, which used the same algorithm as the colour chart to alert patients to contact their surgery if required, as well as presenting readings to clinicians via a web interface. The self-monitoring groups measured their blood pressure twice daily for the first seven days of every month over a 12-month period. 12-month follow-up data were available for 655 participants.

### Included populations

The following additional inclusion criteria were applied to both HOMEBP and TASMNH4 self-monitoring datasets: participants had to have been on a stable dose of their antihypertensive medication for at least 4 weeks, with no clinical indication for adjustment to their medication at their baseline study visit. They had to have submitted more than one month of BP readings during either trial. Any BP readings submitted after a change in medication were removed from the analysis.

### Outcomes

The primary outcome was overall mean change in systolic BP (SBP) and diastolic BP (DBP) per month for each of the included populations. The secondary outcomes were the probability of an individual's BP rising to above threshold at increasing timepoints, the proportions of these readings reflecting true or false positive results, and the variability of systolic and diastolic BP measurements within a set of submitted results per participant.

### Analysis

A mixed effects regression on SBP was performed, using time coefficients to quantify the change in SBP per calendar month. These time coefficients were treated as person-level random effects, so that the mean time coefficient quantified the mean change per month, and the random effects standard deviation (SD) quantified the between-person variation in change per month. The residual SD from the model was interpreted as an estimate of short-term intra-participant BP variability. These regression results were used to estimate probabilities of SBP being above contemporary UK treatments threshold (≥135 mmHg) [15] at subsequent re-measurement; in particular, the residual SD was incorporated to distinguish the probabilities of “true positives” (observed and true SBP≥135 mmHg) and “false positives” (observed SBP≥135 mmHg, but true SBP < 135 mmHg). DBP was analysed similarly (except for the threshold for hypertension being ≥85mmHg). Fitted values and residuals were then calculated for both SBP and DBP models, including the correlations between them, in order to define a bivariate model of BP progression [9,11]. From this, we calculated probabilities of either the SBP being truly above threshold, the DBP being truly above threshold, or both. Any of these three possibilities were defined as hypertension.

Analyses were initially performed on data collected for HOMEBP and then repeated on data from TASMINH4. For the secondary outcome of probability of an individual's BP rising to above threshold at increasing timepoints, modelling was performed using TASMINH4, as it was the larger dataset. Stata 18.0 (StataCorp, 2023) was used for all analyses.

## RESULTS

**FIGURE 1:**
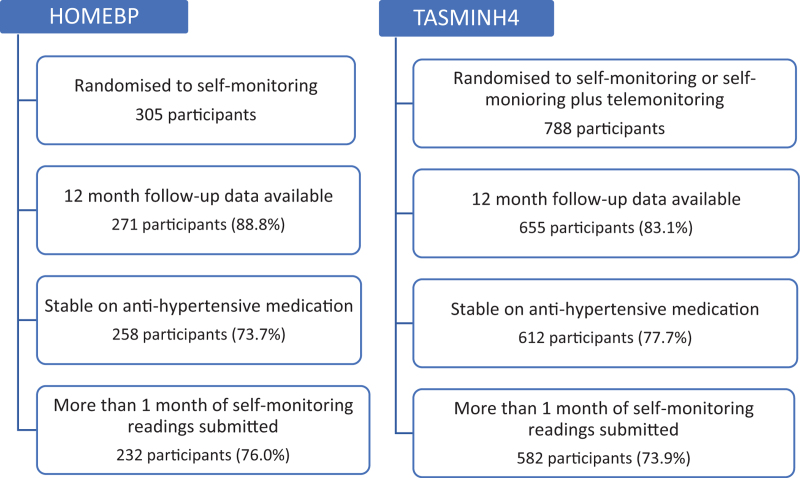
Flow chart documenting the inclusion criteria and the number of individuals included in the study population from each data source.

For HOMEBP, of the 271 participants in the self-monitoring arm, 13 were excluded as not on stable on antihypertensive medication for at least four weeks, 26 were excluded due to insufficient self-monitored readings, leaving 232 (76%) participants who fulfilled the inclusion criteria (Fig. 1). For TASMINH4, of the 655 participants who either undertook self-monitoring or self-monitoring with telemonitoring, 43 were excluded as not on stable antihypertensive medication and 30 were excluded due to insufficient self-monitored readings, leaving 582 (74%) participants who fulfilled the inclusion criteria (Fig. 1).

Table 1 shows the baseline characteristics of eligible participants from both trials which were similar in age [66.4 years (HOMEBP) and 66.9 years (TASMINH4)], mean clinic baseline systolic blood pressures of (134.9 mmHg and 136.8mmHg respectively) and proportion female (51% HOMEBP and 45% TASMINH4). Most participants (94.4% and 96.2%) were of white British ethnicity.

**TABLE 1:** Baseline characteristics

Baseline characteristics	HOME BP self-monitoring (n = 232)	TASMINH4 self-monitoring (n = 582)
Mean age, years (standard deviation)	66.4 (10.0)	66.9 (9.5)
Mean self-monitoring systolic blood pressure, mmHg (standard deviation)	134.9 (11.0)	136.8 (11.5)
Mean self-monitoring diastolic blood pressure, mmHg (standard deviation)	81.2 (8.9)	82.0 (7.6)
Duration of hypertension, years (standard deviation)	11.3 (9.7)	10.2 (8.6)
Female (%)	119 (51)	263 (45)
Ethnicity (%)		
White	219 (94.4)	560 (96.2)
Black /Asian / other	13 (5.6)	22 (3.8)
Past medical history (%)		
Diabetes	19 (8.2)	61 (10.5)
Chronic kidney disease	17 (7.3)	44 (7.6)
Stroke	2 (0.9)	21 (3.6)
Myocardial infarction	5 (2.2)	14 (2.4)
Coronary artery bypass graft, angioplasty, or stent	8 (3.4)	23 (4.0)
Other ongoing medical problem	65 (28.0)	349 (60.0)
Mean body mass index, kg/m^2^ (standard deviation)	29.5 (5.3)	29.6 (7.9)
Number of antihypertensive drugs at baseline (median (interquartile range))	1 (1–2)	2 (1–2)

**FIGURE 2:**
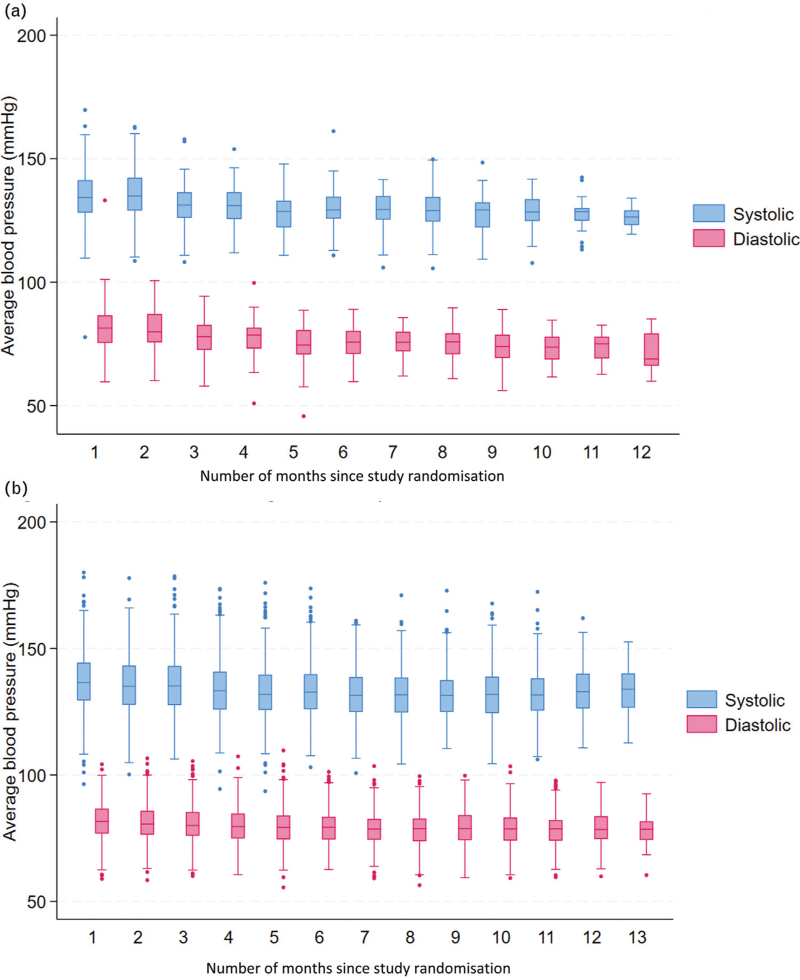
(a) Self-monitoring of blood pressure over time in HOMEBP. (b) Self-monitoring of blood pressure over time in TASMINH4.

Figure 2a and b shows the median, quartiles and range of systolic and diastolic blood pressure over time for HOMEBP and TASMINH4 trials. Observations which fell below quartile 1 minus 1.5 times the interquartile range or above quartile 3 plus 1.5 times the interquartile range are shown as outliers.

### Primary outcome

In HOMEBP, the mean self-monitored SBP at baseline was 134.9 mmHg (SD 11.0 mmHg). The mean change in SBP per month across the whole population was a decrease of 0.1 mmHg. The SD of this change was 0.6 mmHg per month. The mean diastolic BP (DBP) at baseline was 81.2 mmHg (SD 8.9 mmHg). The mean decrease per month was 0.1 mmHg with a SD of 0.2 mmHg. In TASMINH4, the mean self-monitored SBP at baseline was 136.8 mmHg (SD 11.5 mmHg). The mean change over time was a decrease of 0.2 mmHg per month, with a SD of the decrease of 0.7 mmHg. The mean DBP at baseline was 82.0 mmHg (SD of 7.6 mmHg), with a mean decrease over time of 0.1 mmHg and a SD of the decrease of 0.4 mmHg per month.

### Secondary outcomes

#### Variability

In HOMEBP the standard deviation of any 1-week set of SBP measurements submitted by an individual was 4.7 mmHg. In TASMINH4, for any given set of measurements reported by one patient in a calendar month the systolic variability (SD) was 5.1 mmHg. The equivalent variability as measured by SD for DBP was 3.0 mmHg in HOMEBP, and 3.1 mmHg in TASMINH4.

#### Blood pressure control over time

Considering the 582 included participants from TASMINH4, the probability of an individual's SBP rising to above threshold (≥135 mmHg) at increasing timepoints was estimated (Table 2). For a true starting SBP of 130 mmHg, after 6 months the probability that an observed SBP reading was above threshold was 18%, consisting of a 5% probability of being truly above threshold (true positive) and a 14% probability of being truly below threshold (false positive). Therefore, for any reading above threshold, 26% would be true positives (true raised BP), and 74% would be false positives (apparently raised BP due to random variation), odds of 3 : 1 in favour of a raised reading being false positive. After 12 months, the probability of an observed SBP reading being above threshold was 26%, consisting of 17% probability of a true positive and an 9% probability of a false positive (Table 2), now odds of 2 : 1 in favour of a true positive.

**TABLE 2:** Modelled probabilities of observed SBP >135 mmHg using TASMINH4 data, stratified into true and false positives, at 3, 6 and 12 months, expressed as percentages (see text for interpretation)

Time (months)	Observed positives (apparent poor BP control)	True positives (true raised BP)	False positives (falsely raised BP)
			
Percentage of patients predicted to have SBP >135 mmHg			
*If initial SBP=130 mmHg*			
3	15.2%	0.1%	15.0%
6	18.4%	4.7%	13.7%
12	25.6%	16.7%	8.9%
*If initial SBP=125 mmHg*			
3	2.2%	(<0.1%)	2.2%
6	4.1%	0.2%	4.3%
12	11.1%	5.4%	5.7%

Percentage of patients predicted to have DBP >85 mmHg			
*If initial DBP=80 mmHg*			
3	5.2%	(<0.1%)	5.2%
6	6.7%	0.3%	6.3%
12	11.7%	5.4%	6.3%
*If initial DBP=75 mmHg*			
3	0.1%	(<0.1%)	0.1%
6	0.3%	(<0.1%)	0.3%
12	1.8%	0.4%	1.4%

Percentage of patients predicted to have observed hypertension (SBP ≥ 135 mmHg, or DBP ≥ 85 mmHg, or both)			
*If initial BP=130/80 mmHg*			
3	16.6%	0.3%	16.3%
6	18.9%	4.8%	14.1%
12	25.2%	16.0%	9.2%
*If initial BP=125/75 mm Hg*			
3	2.8%	(<0.1%)	2.8%
6	4.7%	0.3%	4.5%
12	11.0%	5.5%	5.5%

Discrepancies in row totals (e.g. 0.3 + 6.3 = 6.7) are due to rounding.

Similarly, the probability of obtaining a true or a false positive result at various time intervals after a true starting SBP of 125 mmHg was estimated (Table 2). At 6 months, only 1 out of every 500 readings would represent a result that was truly above target. At 12 months, there was an 11% probability of observing a BP reading above target, with similar odds of a true or false positive result. Results for DBP can also be seen in Table 2.

Similar results were obtained when the probability of either SBP, DBP or both aspects of an individual's blood pressure rising to ≥135/85 mmHg was estimated (Table 2). For a true initial blood pressure of 130/80 mmHg, after 6 months the probability of observed hypertension was 19%, consisting of a 5% probability of being truly above threshold (true positive) and a 14% probability of a false positive, odds of 3 to 1 in favour of a false positive. After 12 months, the probability of observed hypertension rose to 25%, consisting of 16% probability of a true positive and 9% probability of a false positive, odds of 7 to 4 of a truly raised blood pressure. For an individual with true initial blood pressure of 125/75 mmHg, after 6 months the probability of observed hypertension would be 5% with odds of 1 to 13 in favour of a false positive, and after 12 months, an 11% chance of observed hypertension, with equal odds of true or false positive hypertension.

## DISCUSSION

### Main findings

These results show that across several hundred people with hypertension, self-monitoring BP in primary care with no medication adjustment, there was little monthly change in systolic and diastolic BP readings over a 12-month period, with differences below those measurable by standard equipment, or indeed clinically relevant (less than 1mmHg). However, the trends within individuals varied modestly around this, so that the population mean change reflects a mixture of increasing, decreasing and static BPs. Further, within an individual participant's monthly BP readings there was variability, even over the course of only one week, most likely as a consequence of a combination of measurement error and bio-variability [6,16].

When examining the proportion of BP measurements that were “false positives”, these results found that re-testing BP after a 3 month interval for a patient with previously controlled BP yields almost invariably false positive results, and at 6 months substantially more false positive increases were detected than true positives (odds of 3 : 1). It was only after a 12 month time interval that re-testing was more likely to produce a truly raised BP rather than false positive result. These factors support – and extend to home monitoring – the previous observation that undertaking self-monitoring of BP too frequently once an individual has controlled BP is unlikely to detect clinically relevant changes that would warrant adjustments in management. Careful consideration is still required for patients with a true BP closer to the treatment threshold (e.g. within 5 mmHg for systolic).

### Strengths and limitations

These analyses used data from two large randomized controlled trials of interventions based on self-monitoring of blood pressure, with follow-up for 12 months and similar baseline population characteristics [13,14]. The comparable results found in each dataset add weight to the findings. For these studies, participants were recruited from many general practices across the UK and population characteristics matched those of the hypertensive population including ethnicity (for example white ethnicity 94/96% in TASMINH4/HOMEBP vs 93% in 2021 Office for National Statistics census data) [17]. However the study was not sufficiently large to examine if optimal testing frequencies may be different in specific subgroups. A significant difference between the study populations was the reported number of participants with “other ongoing medical problems”, with HOMEBP reporting 28% and TASMINH4 reporting 60%. It is possible that some of these conditions could have affected the accuracy of BP readings, such as tachyarrhythmias.

The prevalence of co-morbidities was comparable or slightly lower in both TASMINH4 and HOMEBP than would be expected (for example, other studies report 10 - 20% prevalence of diabetes in the hypertensive population) [18], potentially representing a lower baseline level of cardiovascular risk. This should be considered alongside the recommendations from this study, as those with higher cardiovascular risk may warrant more frequent monitoring to ensure aggressive BP control is achieved [15,19]. Similarly, most participants took one or two antihypertensives at baseline and so these results may not hold for those with resistant hypertension.

Patients enrolled in these trials were given proper training in the use of a home blood pressure machine, and were provided with a validated device and an appropriate cuff size. This may not be representative of current general practice, where only 10% of all automated blood pressure monitors using upper arm cuffs available on the global market were validated in one Australian study [20]. However, a recent study found that out of 331 BP monitors used by patients at home in a group of UK practices, 76% passed standard accuracy testing (measured within ±3 mmHg to a reference monitor) [21], rising to 95% if they were within 4 years old and previously validated. This suggests that provided patients are advised regarding appropriate monitors the current study results would apply.

Trial participation might have influenced the findings as those included already had increased motivation relating to controlling their blood pressure, due to their enrolment in a clinical trial [22]. This could have had an impact through improved medication adherence compared to the general hypertensive population, in turn improving blood pressure stability [23]. Furthermore, additional support – including optional lifestyle advice in HOMEBP – might have impacted their BP control via nonpharmacological means. However less than one third chose to receive it suggesting this was not a significant bias [13]. Lifestyle advice was not routinely delivered in TASMINH4, and given the similar results in both datasets it seems unlikely that such advice had a significant impact. Despite this, lifestyle interventions play a key role in maintaining BP and are an important aspect of management [24].

There is a developing body of evidence that high BP variability, both short-term (over 24 h) and long term (over weeks or months) is predictive of cardiovascular outcomes, independent of average BP values [25]. To determine variability multiple repeat BP readings are required, therefore implementation of annual home BP monitoring would reduce data available. However, it is still unclear how variability should be used clinically to improve management, and until this is further understood opting for the proven benefits of reduced monitoring frequency, especially for those with a lower cardiovascular risk profile, would seem most appropriate.

Over the past few years there has been an increased understanding of seasonal variations in blood pressure, with an inverse relationship to outdoor temperatures, with systolic BP being around 5–10 mmHg higher in colder winter months, compared with summer [26]. Given that enrolment in both studies was spread across many months, it is possible some of this impact would have been mitigated, however still needs to be considered as a limitation in this study.

Patients in these studies were treated to the targets outlined in the NICE guideline for hypertension, which are still current best practice in the UK. Since then, new guidelines for the United States and Europe have tended towards lower BP targets [19,27]. Although our quantitative analysis was conducted for the UK thresholds, given that BP is a continuous variable, it is likely that similar results for true and false positive reading frequency would also apply at these lower thresholds, however this was not formally tested.

The conclusions drawn about the frequency of self-monitoring also represent a judgement about the potential benefits and harms of changing antihypertensive therapies based on transiently raised readings, or indeed delaying changes based on readings below the treatment threshold. Further understanding is needed to fully quantify such benefits and harms including the economic costs of taking action more frequently than necessary.

### Comparison to the literature

There has been widespread support for many years for more standardized, evidence-based implementation of self-monitoring of BP. Whilst ambulatory BP monitoring remains the gold standard, its main role is in diagnosis and for those with difficult to control hypertension, due to logistical and practical issues in repeating such measurements frequently. Self-monitoring of BP has been shown to produce results that correlate strongly with ambulatory readings, provided suitable schedules are used, and therefore can be used with confidence for routine monitoring [28]. A survey of 341 UK GPs explored self-monitoring of BP implementation in day-to-day clinical practice, with results ranging from 24% using monthly readings, 34% bi-annual readings and 6% requesting annual readings from patients, demonstrating broad variations between professionals [29]. NICE recommends an “annual review of care for adults with hypertension” [15], but does not comment specifically on how frequently self-monitoring of BP checks should be undertaken. The European Society of Hypertension guidelines from 2010 for home blood pressure monitoring recommend that treated hypertensive patients should perform regular home BP measurements, for example, once or twice a week [30], whereas the 2018 guidelines recommend that once target BP has been reached, an “interval of a few months is reasonable” [31] between hypertension reviews. It is acknowledged that this is an area of debate, which partly justifies these differences, as well as commenting that the higher frequency recommended in 2010 had the additional aim of reinforcing patient compliance with medications. In contrast, other resources from NHS England allude to the benefits of “regular home blood pressure monitoring” in their strategy for cardiovascular disease prevention in primary care [32]. This highlights the lack of consistency across different sources, which maybe partly fuelled by the limited evidence available to date.

Whilst there is currently no consensus in the literature regarding the frequency of self-monitoring for blood pressure, there has been investigation into long-term monitoring of other chronic diseases, such as HbA1c in type 2 diabetes, lipid profiles for hypercholesterolaemia, and albumin-creatinine ratio in diabetic nephropathy. All of these studies have shown that monitoring too frequently significantly increases the risk of detection of false positive changes, leading to undue anxiety for patients, increases in number of follow-up appointments and investigations, and the potential of over-treatment where there is little to gain, but the potential for side effects [7,33]. Buclin *et al.* developed rules to guide frequency of CD4^+^ cell count monitoring in HIV infection. They found that the closer the CD4^+^ cell count was to the threshold where antiretroviral medication should be started, the greater the benefit of increasing frequency of monitoring [34], which is similar to the findings of our analysis.

### Clinical implications

Given that most GPs are using self-monitoring in some form in the management of hypertension, improved understanding to maximize its utility has the potential for significant patient benefit. Firstly, the variability seen between an individual's BP readings reinforces the necessity of using the average from repeated readings across a number of days to provide a more accurate representation of underlying BP. Secondly, adoption of less frequent self-monitoring of BP for well controlled patients would reduce the burden of monitoring for patients, and time spent by healthcare workers reviewing the results and acting on them. A study into the perceived benefits of self-monitoring of BP showed that while some patients found reassurance in regularly seeing their BP was controlled, others found it had the potential for negative emotional implications, such as uncertainty around what should be done with raised readings and worry about if readings were representative. These findings will help clinicians in their education and support of patients undertaking self-monitoring, especially those with health anxieties who may want to self-monitor very frequently, and help them understand that that this might be counterproductive.

One potential downside of adopting recommendations for more infrequent monitoring could be reduced opportunities to check adherence to treatment, with a less frequent patient-prescriber relationship known to play a key role in management plan concordance [35]. However, a Canadian study from 2004 found that clinic follow-up of patients with treated essential hypertension every 6 months was equivalent to every 3 months in relation to adherence to treatment [36], which does not support this concern. Furthermore, patients with well controlled blood pressure are likely to have had a period of more frequent monitoring during medication titration hence previous opportunities to gain from appropriately frequent review.

## CONCLUSION

Given the probabilities of true and false positive results, if blood pressure is controlled well below target then repeating self-monitoring of BP annually is justified. If a patient's baseline BP readings are closer to treatment thresholds, repeating self-monitoring more frequently (for instance 6 monthly) should be considered, especially if they have higher cardiovascular risk due to other factors.

## ACKNOWLEDGEMENTS

The authors acknowledge secretarial support from Lucy Curtin and the original blood pressure monitoring by participants of the HOMEBP and TASMINH4 trials

Sources of funding: This study was funded by the National Institute for Health and Care Research (NIHR) through an academic clinic fellowship to FR. The original studies were funded through NIHR Programme Grants (RP-PG-1211-20001 and RP-PG-1209-10051) and an NIHR Professorship to R.J.M. (NIHR-RP-R2-12-015). R.J.M. receives support from NIHR Oxford and Thames Valley ARC. R.J.M. and L.Y. are NIHR Senior Investigators.

### Conflicts of interest

R.J.M. has worked with Omron to develop a telemonitoring system for which his institution has received consultancy and licencing payments. The other authors declare no relevant conflicts of interest.
